# Type‐I Energy Level Alignment at the PTCDA—Monolayer MoS_2_ Interface Promotes Resonance Energy Transfer and Luminescence Enhancement

**DOI:** 10.1002/advs.202100215

**Published:** 2021-05-05

**Authors:** Soohyung Park, Niklas Mutz, Sergey A. Kovalenko, Thorsten Schultz, Dongguen Shin, Areej Aljarb, Lain‐Jong Li, Vincent Tung, Patrick Amsalem, Emil J. W. List‐Kratochvil, Julia Stähler, Xiaomin Xu, Sylke Blumstengel, Norbert Koch

**Affiliations:** ^1^ Advanced Analysis Center Korea Institute of Science and Technology (KIST) Seoul 02792 South Korea; ^2^ Humboldt‐Universität zu Berlin Institut für Physik & IRIS Adlershof Berlin 12489 Germany; ^3^ Humboldt‐Universität zu Berlin Institut für Chemie Berlin 12489 Germany; ^4^ Helmholtz‐Zentrum für Materialien und Energie GmbH Berlin 12489 Germany; ^5^ Physical Sciences and Engineering King Abdullah University of Science and Technology Thuwal 23955‐6900 Saudi Arabia; ^6^ Department of Mechanical Engineering The University of Hong Kong Pokfulam Road Hong Kong; ^7^ Shenzhen Geim Graphene Center Tsinghua‐Berkeley Shenzhen Institute Tsinghua University Shenzhen 518055 China

**Keywords:** energy level alignment, energy transfer, MoS_2_, organic semiconductors, photoelectron spectroscopy, photoluminescence, transient absorption spectroscopy

## Abstract

Van der Waals heterostructures consisting of 2D semiconductors and conjugated molecules are of increasing interest because of the prospect of a synergistic enhancement of (opto)electronic properties. In particular, perylenetetracarboxylic dianhydride (PTCDA) on monolayer (ML)‐MoS_2_ has been identified as promising candidate and a staggered type‐II energy level alignment and excited state interfacial charge transfer have been proposed. In contrast, it is here found with inverse and direct angle resolved photoelectron spectroscopy that PTCDA/ML‐MoS_2_ supported by insulating sapphire exhibits a straddling type‐I level alignment, with PTCDA having the wider energy gap. Photoluminescence (PL) and sub‐picosecond transient absorption measurements reveal that resonance energy transfer, i.e., electron–hole pair (exciton) transfer, from PTCDA to ML‐MoS_2_ occurs on a sub‐picosecond time scale. This gives rise to an enhanced PL yield from ML‐MoS_2_ in the heterostructure and an according overall modulation of the photoresponse. These results underpin the importance of a precise knowledge of the interfacial electronic structure in order to understand excited state dynamics and to devise reliable design strategies for optimized optoelectronic functionality in van der Waals heterostructures.

## Graphical abstract

The energy level alignment of the van der Waals heterojunction perylenetetracarboxylic dianhydride/monolayer MoS_2_ is determined to be a straddling type‐I. This enables sub‐picosecond time scale resonance energy transfer, i.e., interfacial exciton transfer, and interfacial charge transfer is not supported.

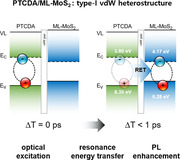

## 1 Introduction

2D transition metal dichalcogenides (TMDCs) are promising next‐generation semiconductors because of their superior electronic and optoelectronic properties originating from their low dimensionality.^[^ ^1^, ^2^, ^3^ ^]^ In the monolayer (ML) regime, these materials with a thickness of only ≈0.7 nm have a direct bandgap and thus strong light‐matter coupling. The lowest energy optical excitations are excitons with typical exciton binding energy in the range of hundreds of meV (depending on the surrounding dielectric media), assuring excitonic luminescence up to room temperature. Furthermore, flat samples with low defect concentration can be prepared, so that charge carrier scattering is low and high carrier mobility of a few hundred cm^2^ V^−1^ s^−1^ have been achieved,^[^ ^4^ ^]^ and superior resistance to short‐channel effects was demonstrated in field‐effect transistors.^[^ ^5^ ^]^ However, the reduced out‐of‐plane size translates into limited optical density. For example, the fraction of photons absorbed by a ML‐TMDC just three atomic layers thick is naturally low [≈5% at the lowest excitonic resonance in monolayer MoS_2_ (ML‐MoS_2_)].

One approach to overcome this problem is the combination of ML‐TMDCs with conjugated organic molecules that also feature strong light‐matter coupling. If properly designed, molecular layers can enhance and spectrally tune light absorption and emission of such heterostructures, often of the van der Waals type.^[^ ^6^ ^]^ Investigations of the photoexcitation dynamics in organic molecule/TMDC heterostructures have mostly focused on optically induced charge‐transfer (CT) processes. When these structures exhibit a staggered type‐II energy level alignment, excitons are dissociated and electrons and holes are spatially separated in the two material components. Ultrafast (<150 fs) CT has been observed in such configurations, and these were employed in hybrid photodetectors, where the absorption of light in the organic layer was shown to enhance and spectrally extend the photoresponse.^[^ ^7^, ^8^, ^9^, ^10^, ^11^ ^]^

Despite the above mentioned strong light‐matter interaction, ML‐TMDCs generally suffer from comparably low photoluminescence (PL) yield at room temperature. Besides meticulous reduction of structural defects in the monolayer, the combination with organic molecules can also provide a solution here. One approach consists of the exploitation of resonance energy transfer (RET), which to date has been realized and studied to a lesser extent for organic molecule/TMDC heterostructures. One such example is the study of energy transfer between a molecular J‐aggregate and MoS_2_,^[^ ^12^ ^]^ but an enhancement of the PL from ML‐MoS_2_ was not observed. Rather, owing to the type‐II energy level alignment of this hybrid structure, exciton dissociation, and PL quenching of both components was concluded on. On the other hand, significant PL enhancement was reported for a hybrid structure composed of perylenetetracarboxylic dianhydride (PTCDA) and ML‐MoS_2_.^[^ ^13^ ^]^ Based on separately determined values of the electron affinity and ionization energy of the individual materials and from theoretical modeling^[^ ^14^ ^]^ a type‐II interfacial level alignment was suggested, with an offset between the highest occupied molecular orbital (HOMO) level and the ML‐MoS_2_ valence band maximum (VBM) of 0.5 eV. The increase in PL was then explained by excited state CT from MoS_2_ to PTCDA, a slight hybridization of PTCDA and ML‐MoS_2_ orbitals, and an improved crystallinity of the PTCDA film grown on MoS_2_. In another example, a MoS_2_/PTCDA heterojunction was shown to be able to simulate the function of a synapse, and based on this observation, its use as a building block in neuromorphic computational devices was suggested.^[^ ^15^ ^]^ To explain the electric characteristics of that heterojunction, CT across the assumed type‐II interface was again invoked.^[^ ^15^ ^]^ Also recently, an experimental study of the PTCDA/ML‐MoS_2_ interface claimed a huge energy offset of 1.18 eV between the HOMO level of PTCDA and the VBM of ML‐MoS_2_, implying a pronounced type‐II level situation, and accordingly efficient interfacial electron transfer subsequent to optical excitation was concluded on.^[^ ^16^ ^]^ In this latter work, however, the peak maximum of the HOMO photoemission feature was used to construct the energy level diagram, instead of the low binding energy HOMO manifold onset, which is the relevant energy for charge carrier transfer.^[^ ^17^, ^18^ ^]^

The above examples show that the electronic structure and energy level alignment between organic molecules and ML‐TMDCs are of utmost importance, as they govern ground‐ and excited‐state CT interactions, which lie at the heart of the functionality of a device. The widespread attraction of ML‐TMCD/PTCDA for various applications,^[^ ^13^, ^15^, ^19^, ^20^ ^]^ has prompted us to investigate the electronic structure of the PTCDA/ML‐MoS_2_ interface in detail and to reconsider photoinduced CT and RET processes occurring therein. This is partly motivated by the fact that the prediction of the energy level alignment based on electronic structure data of the individual components is highly problematic, because the energy gap as well ground‐state CT of ML‐TMDCs are strongly influenced by the supporting substrates, as recently demonstrated.^[^ ^21^ ^]^ To obtain the intrinsic properties of the PTCDA/ML‐MoS_2_ interface a pertinent study on an inert supporting substrate, insulating sapphire in this study, is required.^[^ ^21^ ^]^ Angle‐resolved direct and inverse photoemission spectroscopy (ARPES and ARIPES) reveal that the energy level alignment is actually straddled type‐I, with PTCDA being the wider gap component. Ground‐ and excited‐state CT, and thus charge separation, are energetically not possible. Continuous‐wave and time‐resolved PL measurements show efficient transfer of electron–hole pairs, i.e., excitons, from PTCDA to ML‐MoS_2_. This process is identified as the origin of the enhancement of the TMDC PL yield in the heterostructure. Transient absorption (TA) spectroscopy indicates that the RET between MoS_2_ and PTCDA occurs on a sub‐picosecond time scale and competes thus efficiently with radiative and nonradiative recombination processes in the donor material. RET in type‐I TMDC/molecule heterostructures can thus provide an effective route toward improving the PL characteristics of ML‐TMDCs.

## 2 Results and Discussion

**Figure 1:**
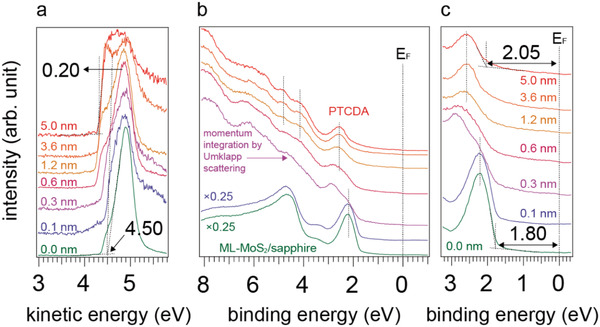
Secondary electron cutoff (SECO) and valence band (VB) spectra of a,b) ML‐MoS_2_/sapphire with increasing PTCDA coverage as noted next to the spectra. c) VB spectra near the Fermi level to determine the onset of the valence band maximum (VBM) and HOMO onset. All spectra were recorded in the surface normal direction corresponding to the Γ point of the ML‐MoS_2_ Brillouin zone.

ARPES measurements for incrementally increased PTCDA coverage on ML‐MoS_2_, shown in **Figure** **1**, allow the determination of the energy level alignment. All spectra were recorded at the Γ point of the Brillouin zone (BZ) to obtain reliable secondary electron cutoff (SECO) spectra and for improved signal‐to‐noise‐ratio (which is a little lower at the K point). Further methodological details are explained in a previous work.^[^ ^21^ ^]^ In Figure 1a, the onset of the SECO provides a work function (Φ) value of 4.50 eV for the bare ML‐MoS_2_ surface. Upon stepwise increase of the PTCDA layer thickness, Φ shifts toward lower values and quickly saturates at 4.30 eV. Since our further experimental results (vide infra) point toward a van der Waals type interaction at the interface, we assign this small Φ change not to interfacial charge transfer but to a surface electron push‐back effect, in analogy to what has been observed for the deposition of molecules onto various inert metal oxide surfaces, which is characteristic for interfaces formed by weakly interacting compounds.^[^ ^22^, ^23^, ^24^, ^25^ ^]^ From Figure 1b,c, it is evident that the VBM of MoS_2_ maintains its original energy position at 1.80 eV below the Fermi level (set to zero), with only a very slight broadening for a PTCDA nominal thickness of 0.1 nm. For 0.3 nm PTCDA thickness (slightly less than a monolayer), a pronounced change of the valence band shape is observed. Similar sudden spectral changes of van der Waals materials surfaces upon molecular deposition or adsorbed contaminants were observed before, but remained unexplained.^[^ ^26^, ^27^, ^28^ ^]^

**Figure 2:**
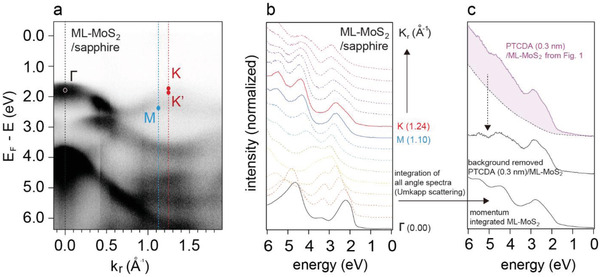
ARPES spectra of bare ML‐MoS_2_ on sapphire plotted a) as band structure and b) as energy distribution curves (EDC) as a function of energy and momentum. c) Comparison of an angle‐integrated EDC of bare ML‐MoS_2_ (bottom), and the EDCs of PTCDA (0.3 nm)/ML‐MoS_2_/sapphire with and without background subtraction (middle/top) at the Γ point.

To uncover the origin of the abrupt spectral change, the ARPES spectra of bare ML‐MoS_2_ are plotted in conventional band structure format and as the corresponding energy distribution curves (EDCs) as a function of the momentum in **Figure** **2**a,b. The dispersion of the momentum‐dependent spectral distribution is in good agreement with the band structure obtained by density functional theory and previous ARPES measurements.^[^ ^29^, ^30^, ^31^ ^]^ In contrast, the PTCDA (0.3 nm)/ML‐MoS_2_ spectra are independent of momentum (see Figure S1, Supporting Information). Considering the low coverage of 0.3 nm and the photoelectron cross‐section of PTCDA, the contribution of the molecules to the valence features are barely discernable.^[^ ^32^ ^]^ Therefore, we assign the abrupt spectral change to surface‐electron elastic and inelastic scattering of electrons emitted from ML‐MoS_2_ by the molecular overlayer. Inelastic scattering generates a background signal gradually increasing with lower kinetic energy, as shown by the dashed line in the top spectrum of Figure 2c. Elastic scattering leads to momentum integration of the ML‐MoS_2_ photoelectrons over the whole BZ as photoelectrons gain crystal momentum from the PTCDA lattice when passing through the organic layer. The latter process is also known as surface Umklapp scattering,^[^ ^33^, ^34^, ^35^ ^]^ or general photoelectron scattering processes that change the original momentum can give rise to a loss of momentum‐dependence in ARPES. This is strongly suggested by comparing the spectra of the momentum (angle)‐integrated bare ML‐MoS_2_ spectrum (the sum of all EDCs from Figure 2b) and the background‐corrected spectrum for PTCDA (0.3 nm)/ML‐MoS_2_, which are very similar aside from small differences due to the adsorbed PTCDA. Therefore, the observed changes of the MoS_2_ valence features upon deposition of PTCDA are thus not due to differential spectral shifts or orbital hybridization, but they are due to the photoelectron surface‐scattering effect. A notable modification of the ML‐MoS_2_ valence band induced by PTCDA can thus be ruled out.

With further increase of the PTCDA coverage up to 5 nm, the spectral weight of ML‐MoS_2_ and PTCDA gradually changes until only the features of PTCDA are visible. No spectral shifts of the PTCDA energy levels are observed (see dashed lines in Figure 1b,c) and the spectra are weighted superpositions of the two individual components. This further implies weak interaction of the van der Waals type, and the absence of ground state CT or sizeable orbital hybridization.

**Figure 3:**
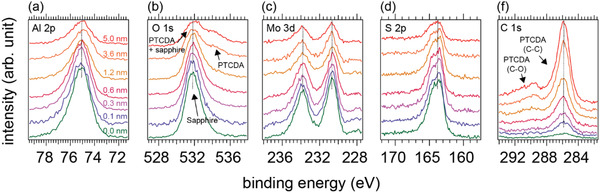
Core‐level spectra of ML‐MoS_2_/sapphire with increasing nominal PTCDA coverage as indicated next to the spectra.

The core‐level spectra measured for increasing PTCDA coverage on ML‐MoS_2_ and shown in **Figure** **3** provide further support for van der Waals bonding at this interface. The Al 2p and O 1s (for zero coverage) levels are from the sapphire substrate and the Mo 3d and S 2p levels from ML‐MoS_2_. As the coverage of PTCDA is incrementally increased, all core levels related to sapphire and ML‐MoS_2_ maintain the same peak position with a gradual intensity decrease due to signal attenuation by the PTCDA overlayer. Additional core‐level features originating from PTCDA arise, which can be assigned to its O 1s and C 1s levels according to Wang et al.^[^ ^28^ ^]^ The absence of core‐level shifts further confirms weak electronic interaction at the PTCDA/ML‐MoS_2_ interface.

**Figure 4:**
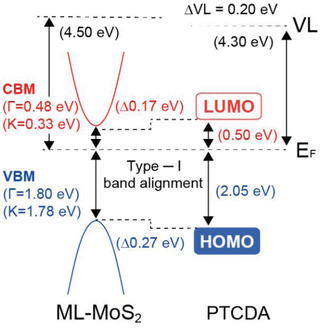
Schematic energy level alignment for PTDCA/ML‐MoS_2_ on an insulating sapphire substrate, as derived from ARPES and ARIPES measurements. The values for the ML‐MoS_2_ CBM and VBM are given for both the Γ and K points of the BZ.

**Figure** **4** depicts the type‐I energy level alignment at the PTCDA/MoS_2_ heterostructure derived from ARPES and ARIPES as follows: i) The VBM of ML‐MoS_2_ at the Γ point of 1.80 eV was obtained from the onset of the valence band of the bottom spectrum in Figure 1c. ii) As the interface is of van der Waals type, band structure renormalization of ML‐MoS_2_ is not expected to be significant. iii) Based on this, the global VBM and conduction band minimum (CBM), which are located at the K point of the BZ, are given with respect to the VBM at the Γ point, where the energy difference between the Γ and K points (0.02 eV for VBM and 0.15 eV for CBM) was taken from previous work.^[^ ^36^ ^]^ iv) The lowest unoccupied molecular orbital (LUMO) level of PTCDA was directly measured by ARIPES (see Figure S2, Supporting Information), and the HOMO level onset was determined from the top spectrum in Figure 1c. We thus observe from Figure 4 that the PTCDA levels straddle those of ML‐MoS_2_ in a type‐I manner, yielding values for the energy offsets of the occupied and unoccupied levels of 270 and 170 meV, respectively. This has notable consequences for charge carriers and optical excitations of the heterostructure. Individual electrons (holes) injected into the PTCDA LUMO (HOMO) can be transferred to the lower energy gap ML‐MoS_2_. For optically excited bound electron–hole pairs, i.e., excitons, dissociation, and interfacial charge separation is energetically not favored. However, the type‐I interface facilitates the transfer of excitons via RET and possibly an enhancement of the MoS_2_ PL yield, as in fact demonstrated in the following.

**Figure 5:**
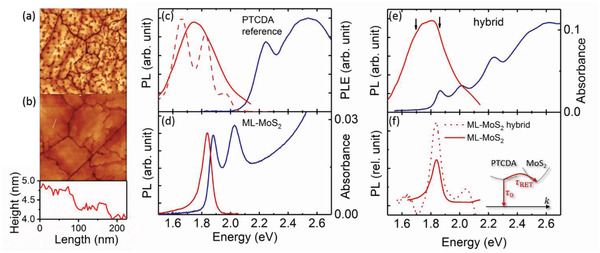
AFM images of the PTCDA reference a) and hybrid sample b). The nominal thicknesses of the PTCDA layers are 18 and 6 nm, respectively. The height scan was taken along the white line in b). The scan size is 2 × 2 µm^2^. c) PL (red) and PLE (blue) spectra of the thick PTCDA film (18 nm) on ML‐MoS_2_ (PTCDA reference). The PLE spectrum was recorded setting the detection energy to 1.77 eV. The sample temperature was 293 K (solid lines) and 4.5 K (dashed line). d) PL (red) and absorbance (blue) of bare ML‐MoS_2_ on Al_2_O_3_. e) PL (red) and absorbance (blue) spectra of the thin PTCDA film (6 nm) on ML‐MoS_2_ (hybrid). f) Comparison of the PL yield of MoS_2_ of the bare ML‐MoS_2_ (solid line) and the hybrid (dashed line) sample. The latter is obtained by calculating the difference between the PL of the hybrid and that of the PTCDA reference scaled to the intensity of the hybrid PL at 1.65 eV, where MoS_2_ does not emit to take the different layer thicknesses into account. The inset shows the different decay channels of PTCDA molecules in the hybrid sample. *τ*_0_ is the decay time of PTCDA due to radiative and nonradiative recombination and *τ*_RET_ the RET time constant. The PL in all experiments was excited at 2.21 eV.

For PL investigations, we compare ML‐MoS_2_ without and with PTCDA deposited on top. The corresponding three samples are referred to as ML‐MoS_2_, hybrid (with 6 nm PTCDA), and PTCDA reference (with 18 nm PTDCA). The PL properties of PTCDA are known to be strongly influenced by the structure and morphology of the thin films, which, in turn, depend on the deposition conditions and, in particular, on the substrate.^[^ ^37^, ^38^ ^]^ To minimize such influences, the 18 nm thick PTCDA film on ML‐MoS_2_ was chosen as a reference because its spectra are dominated by the contributions of molecular layers far from the interface. The arrangement of the molecules is the same for both PTCDA layers, as seen from atomic force microscopy (AFM) images of the hybrid and the PTCDA reference sample depicted in **Figure** **5**a,b. The morphology is typical for PTCDA on flat weakly interaction substrates, and the measured step height of 0.35± 0.05 nm corresponds closely to the spacing of the (102) plane of the PTCDA crystal.^[^ ^39^, ^40^ ^]^ Whether the *α* or *β* phase of the PTCDA crystal prevails in the thin film cannot be determined from the AFM images because the layer spacing is nearly identical. Importantly, in both polymorphs, the PTCDA molecules lie parallel to the substrate surface in a herringbone arrangement within the (102) plane.^[^ ^39^, ^40^ ^]^ Figure 5c,d compares the optical spectra of bare ML‐MoS_2_ and the PTCDA reference sample. The photoluminescence excitation (PLE) spectrum of the PTCDA reference reflects the absorption of the molecular layer. It is independent of the detection energy and shows the known features of thick polycrystalline PTCDA films.^[^ ^41^, ^42^ ^]^ The origin of the weaker and narrower feature with a maximum at ≈2.2 eV and the higher‐energy broad band with a maximum at 2.6 eV are still debated. A recent study suggested that the broad absorption band at 2.4–3.1 eV is due to a manifold comprising the upper bright Frenkel and various CT states, whereas the lower energy state is predominantly of Frenkel character.^[^ ^43^ ^]^ The PL spectrum is broad and unstructured at room temperature. Lowering the temperature to 4.5 K reveals that the emission does not stem from a single state. Two distinct features emerge with maxima at 1.66 and 1.82 eV, as well as a shoulder at ≈1.95 eV. The two dominant features are often labeled in literature as E‐band and Y‐band, and are assigned to an excimer and to excitons with mixed Frenkel and CT character, respectively,^[^ ^37^, ^44^ ^]^ and their relative intensity is known to depend on the crystalline properties of the thin film.^[^ ^45^ ^]^ The higher‐energy shoulder has been assigned to a nonrelaxed charge transfer exciton transition between stacked molecules in different unit cells.^[^ ^45^ ^]^ The absorption spectrum of the bare ML‐MoS_2_ shows the A and B excitonic transitions at 1.88 and 2.02 eV, respectively.^[^ ^46^, ^47^ ^]^ The PL originates from the A exciton only. The optical gap of PTCDA corresponds to the lowest energy peak of the PLE, and the absorption peak for ML‐MoS_2_. The difference between the energy gap (single particle gap) derived from ARPES and ARIPES and the optical gap yields exciton binding energies of 310 meV for PTCDA and 230 meV for ML‐MoS_2_, in agreement with previous reports.^[^ ^36^, ^48^ ^]^ Most importantly, the prerequisite for RET, namely spectral overlap between the PL of PTCDA and the absorption of ML‐MoS_2_, is met.

**Figure 6:**
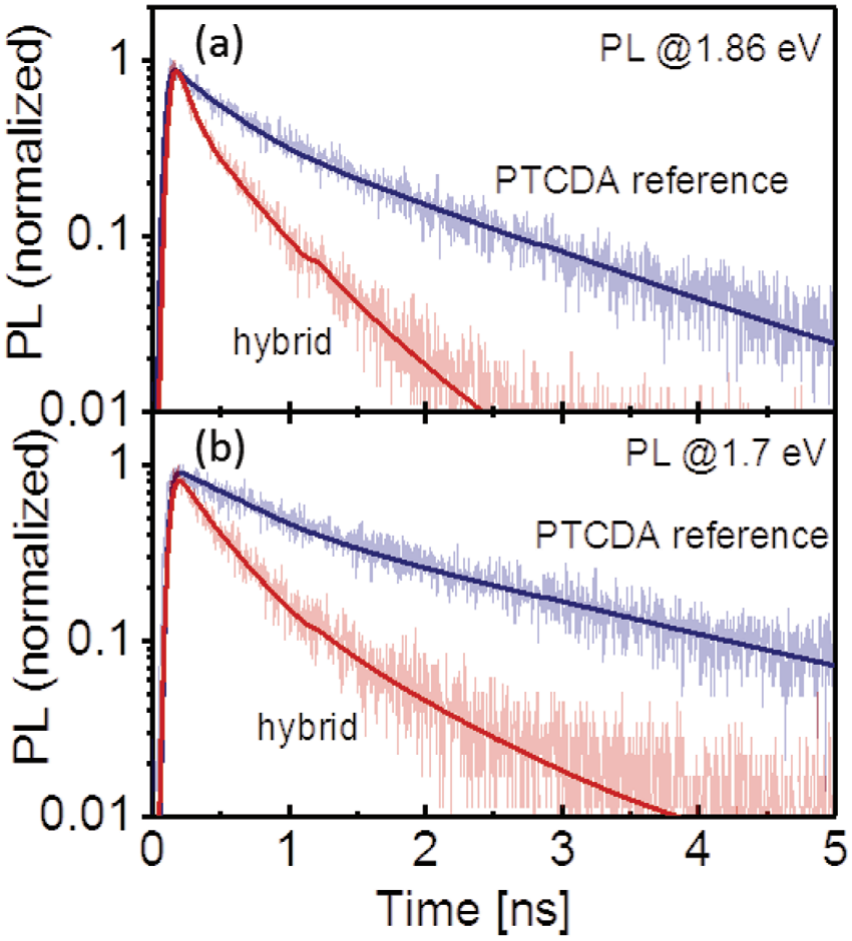
a,b) PL transients of PTCDA in the PTCDA reference and hybrid sample recorded at 1.86 eV a) and 1.7 eV b). The fits were obtained by convoluting biexponential decay laws with the instrument response function. The detection energies are marked by arrows in Figure 5e. The PL in all experiments was excited at 2.21 eV.

The absorption and PL spectra of the hybrid structure, depicted in Figure 5e, are superpositions of the respective spectra of the individual components. Although the PL spectra overlap, and despite the larger thickness of the PTCDA layer (6 nm) than the ML‐MoS_2_, the PL peak of the latter is clearly recognizable. The evidence of RET is provided by time‐resolved PL measurements (**Figure** **6**a,b). The PL decay curves of the hybrid and PTCDA reference samples were recorded at detection energies above (1.86 eV) and below (1.7 eV) the ML‐MoS_2_ PL band, i.e., probing only the PTCDA PL. The comparison shows that the PL decay in the hybrid as well as in the PTCDA reference is slower at lower energies. In the PTCDA reference sample, the mean PL lifetime 〈*τ*〉 increases from 1.15 ns at 1.86 eV to 1.95 ns at 1.7 eV. 〈*τ*〉 is thereby defined as ⟨τ⟩=∫t·I(t)dt/∫I(t)dt with the decay transient *I*(*t*). The dependence of the PTCDA reference PL lifetime on the emission energy can be attributed to the fact that different excited species contribute to the PL spectrum, as discussed above. Their relative weight changes with the emission energy, as does the mean decay time. PL transients recorded at 4.5 K (Supporting Information) and literature PL measurements at 77 K suggest that the lifetime of the E‐band is considerably longer than that of the Y‐band.^[^ ^44^ ^]^ However, independent of the emission energy, a clear shortening of the PL lifetime is observed in the hybrid structure, which is a clear indication of an additional decay channel for PTCDA excitations due to RET (inset of Figure 5f), as charge transfer is not supported by the type‐I level alignment (see Figure 4). The PL decay is nonexponential over the entire spectral range. The origin lies in part in the different species contributing to the PL as well as to the RET process itself, as discussed below. A lower bound for the efficiency of RET given by *η*_RET_ = (*I*_0_ − *I*_h_) /*I*_0_, where *I*_h_ and *I*_0_ are the PTCDA PL yields of the hybrid and PTCDA reference samples, respectively, is obtained by integrating the normalized PL transients after deconvolution with the system response function. Independent of the emission energy, *η*_RET_ ≈ 0.7 is obtained. This means that at least 70% of the excitons generated in the 6 nm thick PTCDA layer are transferred to ML‐MoS_2_. The value represents a lower bound since in the PTCDA reference sample energy transfer is not entirely negligible as discussed in more detail below. Most importantly, the excitation energy drained from PTCDA generates PL in MoS_2_. This is shown in Figure 5f, which compares the PL intensity of bare ML‐MoS_2_ and that of the monolayer in the hybrid sample. Due to RET, the PL yield of ML‐MoS_2_ in the hybrid is enhanced by a factor of ≈2. PTCDA PL decay transients recorded 4.5 K indicate that RET is switched off for the E‐band while the efficiency drops to *η*_RET_ ≈ 0.3 for the Y‐band at low temperature (see Figure S3, Supporting Information). The Varshni shift, i.e., the increase of the optical gap upon lowering the temperature from 293 to 4.5 K, is about 80 meV in MoS_2_,^[^ ^49^ ^]^ while in the same temperature range, the PTCDA PL spectrum narrows but does not spectrally shift (see Figure 5c). As a result, the spectral overlap between the MoS_2_ absorption and the PTCDA PL, which is a prerequisite for RET, becomes smaller for the Y band and vanishes for the E band. The observed drop of the RET efficiency with temperature provides thus further evidence that RET is the relevant transfer process that governs the PTCDA PL decay dynamics in the hybrid sample at room temperature.

**Figure 7:**
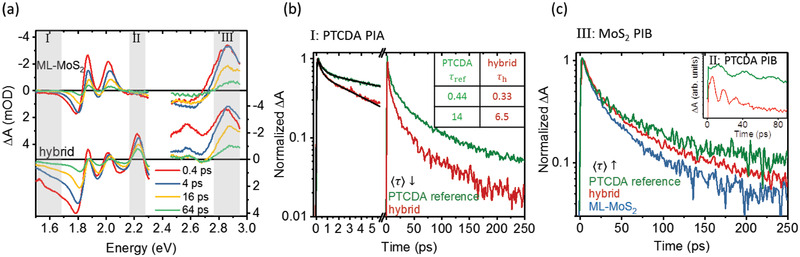
a) Transient absorption (TA) spectra of bare ML‐MoS_2_ and the hybrid structure at different delay times. The spectra are inverted for better comparability with the absorption spectra. The data points in the spectral range of the pump pulse at 2.43 eV are removed. The spectral bands for analysis of the kinetics are highlighted by the gray shaded areas: region I for the PTCDA PIA, region II for the PTCDA PIB, and region III for the ML‐MoS_2_ PIB. b) Kinetics of the PIA band between 1.49 and 1.61 eV reflecting the decay of the PTCDA excited‐state absorption. The table gives the time constants (in ps) for delay times between 0 and 5.7 ps. They are derived from fits of the decay transients in the respective time window which are obtained by convoluting biexponential decay laws with the system response function (solid lines). c) Kinetics of the PIB band between 2.75 and 2.95 eV dominated by the MoS_2_ C exciton resonance. The inset shows the kinetics of the PTCDA PIB band between 2.17 and 2.27 eV reflecting the ground‐state recovery of PTCDA.

The kinetics of RET are determined by transient absorption (TA) spectroscopy. The TA spectra of bare ML‐MoS_2_ and the hybrid structure for different delay times are depicted in **Figure** **7**a. The TA spectra of bare ML‐MoS_2_ are comprised of dispersive features due to the excitonic A, B, and C resonances. The shape and the evolution of the spectra resemble literature data.^[^ ^50^, ^51^ ^]^ All features are already present at early pump–probe delays (100 fs). The evolution of the spectra is independent of the photon energy of the pump pulse (2.2 and 2.43 eV). Such independence was observed previously employing pump pulses in the range of 1.88–3.06 eV.^[^ ^50^ ^]^ The bleaching and shifting of the three excitonic resonances leading to the dispersive shape of the signals are due to phase‐space filling, bandgap renormalization, and Coulomb screening due to excited carriers.^[^ ^50^, ^51^, ^52^, ^53^, ^54^ ^]^ These spectral features are also clearly recognizable in the TA spectra of the hybrid structure, where they are superimposed on the photoinduced bleaching (PIB) and photoinduced absorption (PIA) of PTCDA above and below 2.1 eV, respectively. The negative PIB features at 2.2 and 2.6 eV are assigned to ground‐state bleaching because they resemble the linear absorption spectrum in accordance with the TA spectra of a thick PTCDA film on sapphire substrates reported in literature.^[^ ^55^ ^]^ The PIA band is due to excited‐state absorption.^[^ ^55^ ^]^ The spectra of the PTCDA reference resemble those of the hybrid sample, but with the spectral weight shifted toward PTCDA (see the Supporting Information). The spectral bands defined for analysis of the transient behavior of the PTCDA PIA (region I), the PTCDA PIB (region II), and the PIB signal related to the ML‐MoS_2_ C exciton (region III) are indicated in Figure 7a. The ML‐MoS_2_ A and B excitons are not included in the analysis because of the large uncertainty due to the spectral overlap with the PTCDA excited‐state absorption in the hybrid structure.

The decay of the PTCDA PIA signal (Figure 7b), reflecting the depopulation of the excited state, is nonexponential and shortens in the hybrid structure compared to the PTCDA reference. For a thick (≈30 nm) polycrystalline PTCDA film on sapphire, a nonexponential behavior was observed in a time window 0 … 450 ps at comparable excitation densities (≈7.5  ×  10^19^ cm^3^) and explained by diffusion‐assisted bimolecular exciton recombination.^[^ ^55^ ^]^ An exciton diffusion length of *l*_D_ = 61 nm along the stacking direction of the molecules was derived. Other studies report *l*_D_ = 22 nm in *α*‐PTCDA single crystals based on PL measurements.^[^ ^56^ ^]^ The large *l*_D_ suggests that the RET from PTCDA to ML‐MoS_2_ is not a single‐step process but one assisted by diffusion and, furthermore, that even in the PTCDA reference (18 nm PTCDA on ML‐MoS_2_), energy transfer is not entirely negligible. RET can be mediated by dipole–dipole interaction, i.e., Förster transfer, and exchange interaction, i.e., Dexter transfer. The parallel orientation of the transition dipole moments of the flat‐on adsorbed PTCDA molecules and ML‐MoS_2_ is ideal for Förster transfer. Furthermore, this adsorption geometry also maximizes the wave‐function overlap between PTCDA and ML‐MoS_2_ that is essential for Dexter transfer. The dependence of the RET rate constant on the distance *d* between the molecules and the ML‐MoS_2_, namely ≈ *d* ^−4^ for a Förster‐type interaction in the present 2D setting, and ≈ *e* ^−*d*^ for Dexter transfer, results in a distribution of RET rates and thus, like diffusion, to nonexponential decay. The disentanglement of the individual contributions to the total energy transfer by fitting the decay transients has not been undertaken as reliable results are not expected because of the large number of parameters and the limited data set. Furthermore, the dipole approximation is not accurate for molecules near the interface and thus the distance dependence of the RET rate constant deviates from the Förster law. Therefore, only a qualitative picture of the energy transfer process will be drawn. Nevertheless, an estimate of the characteristic RET time constant *τ*_RET_ can be derived for molecules in immediate proximity to the ML‐MoS_2_, because their contribution decays at the earliest times. According to literature, the PTCDA PIA signal of thick polycrystalline PTCDA on sapphire does not decay in a time window between 0 and 600 fs at comparable excitation densities.^[^ ^55^ ^]^ Thus, the initial short decay components of the hybrid structure [(0.3± 0.1)ps] and the PTCDA reference [(0.4± 0.1)ps] obtained from a fit with a simple biexponential decay law (see inset‐table of Figure 7b) reflect the depopulation of the PTCDA excited state due to a single‐step RET, which occurs consequently on a sub‐ps time scale. It should be recalled here that also the PTCDA reference sample contains ML‐MoS_2_ and therefore, also in this sample there are molecules that decay on such short time scale due to the single‐step RET to ML‐MoS_2_. The fraction of these molecules is, however, much smaller than in the hybrid sample. Nevertheless, the contribution of these fast‐decaying molecules is also detectable in the PTCDA PIA kinetics of the PTCDA reference sample. Single‐step RET to ML‐MoS_2_ is apparently much faster than the radiative and nonradiative decay in PTCDA, and therefore RET is the dominating de‐excitation channel for molecules in close proximity to ML‐MoS_2_. For molecules further away from the interface, but close enough for a single‐step RET, *τ*_RET_ increases according to the distance dependence discussed before while diffusion‐assisted RET from molecules still further away occurs on still longer time scales. The overall slower decay of the PIA signal of the PTCDA reference sample is due to the large fraction of molecules which do not or only via slow diffusion‐assisted RET engage in energy transfer. A complementary analysis of the ground‐state bleaching recovery dynamics in PTCDA (region II) is hampered by the superposition of oscillations due to the excitation of acoustic phonons (Figure 7c).^[^ ^57^, ^58^ ^]^ The resultant shift of the transition energy leads to a modulation of the decay signal with a period *T* proportional to the film thickness *d*. The sound velocity within PTCDA can thus be derived as $v_{S}=\frac{4d}{T}\;\approx 2.2\times 10^{5}\mathrm{cm}\;s$ ^−1^. Finally, the increase of the decay time of the MoS_2_ C exciton in the hybrid structure and the PTCDA reference clearly reflects the supply of excitons from the molecular layer to ML‐MoS_2_ by RET (Figure 7c).

## 3 Conclusions

We demonstrate by direct and inverse photoelectron spectroscopy that the energy level alignment at the interface of PTCDA and ML‐MoS_2_ corresponds to a type‐I heterojunction, where the PTCDA frontier levels straddle those of ML‐MoS_2_ by well over 100 meV. This alignment does not facilitate ground‐ or excited state charge transfer or exciton dissociation. Furthermore, neither core level nor valence photoelectron spectra yield any indication for a hybridization of the PTCDA orbitals with those of MoS_2_. This is in contrast to the previous understanding that hybridization and excited‐state charge transfer are the origin of the intriguing optoelectronic performance of PTCDA/ML‐MoS_2_ heterostructures. Photoluminescence and transient absorption spectroscopy reveal, instead, efficient exciton transfer, i.e., RET, from PTCDA to ML‐MoS_2_. For molecules in close proximity to the interface, the transfer occurs within the first picosecond, which is much shorter than radiative and nonradiative recombination in the donating PTCDA. Efficient RET provides thus the route to the enhancement of the PL yield and the modulation of the photoresponse of ML‐MoS_2_. The present findings underpin that a precise knowledge of the electronic structure at the interface between organic molecules and 2D semiconductors is the key for understanding excitation dynamics and optoelectronic function of van der Waals heterostructures.

## 4 Experimental Section

##### Sample Preparation

ML‐MoS_2_ was grown on sapphire via chemical vapor deposition as explained previously.^[^ ^36^, ^59^ ^]^ Prior to the ARPES and X‐ray photoelectron spectroscopy (XPS) measurements, the ML‐MoS_2_ was annealed at 300 °C for ≈12 h to remove the contamination and residual PMMA in the interconnected in situ preparation chamber (10^−9^ mbar). The PTCDA (Sigma‐Aldrich, 97% purity) was outgassed overnight in a preparation chamber to remove unwanted impurities before deposition, and it was deposited on the ML‐MoS_2_ stepwise by monitoring the nominal evaporation speed via quartz crystal microbalance measurement.

##### Photoemission Measurements

ARPES and XPS spectra were obtained using a hemispherical electron analyzer (PHOIBOS‐100, SPECS) using Mg K*α* (1253.6 eV) and He І*α* (21.22 eV) as excitation sources, respectively. ML‐MoS_2_/sapphire was electrically grounded by using mechanical metal contacts at the edges to avoid the sample charging. The continuous coverage of the sample with ML‐MoS2 (see Figure S5 in the Supporting Information) warrant sufficient current percolation paths. Furthermore, a thin (350 nm) Al filter was applied in front of the helium discharge lamp to attenuate the light intensity (reduced by 94%) to prevent sample charging and damage of the organic film under the photon beam. ARIPES spectra were recorded in the isochromat mode using a combination of a BaO‐cathode‐based low‐energy electron gun and an optical bandpass filter consisting of SrF_2_ and NaCl. The energy reference and resolution for ARPES and ARIPES were determined by measuring the Fermi level of a clean Au (111) single crystal. The total resolution was 0.09 and 0.30 eV (after deconvolution; details in Ref. ^[^ ^23^ ^]^) for ARPES and ARIPES, respectively. For XPS, the full width half maximum of the Au 4f core level was used to estimate the resolution of 0.75 eV.

##### PL Spectroscopy

The experiments were performed at a *μ*‐PL set‐up using a laser diode (Picoquant) emitting at 2.21 eV as excitation source. The average intensity was 70 W cm^−2^ for cw and time‐resolved measurements (80 MHz pulse repetition rate, pulse length 70 ps FWHM). This corresponds to ≈2.5  ×  10^12^ photons cm^−2^ per pulse. PL transients were recorded employing time‐correlated single photon counting.

##### TA Spectroscopy

The TA setup with applications has been described elsewhere.^[^ ^60^, ^61^, ^62^ ^]^ Briefly, TA spectra are recorded with 8–12 forward and backward pump–probe scans, with 0.02, 0.2, and 2 ps steps. The pump–probe pulse cross‐correlation is 100 fs. The TA signal consists of three contributions: negative bleach, negative stimulated emission (SE), and positive excited‐state absorption (ESA). The spectra are measured at different pump photon energies (2.2 and 2.43 eV) and pump fluences. Since the spectra have been found to be independent of the photon energy and the pump fluence in the studied range, only spectra obtained at a pump photon energy of 2.43 eV and a fluence of 150 µJ cm^−2^ are reported. The pump fluence translates into an incident photon density of ≈4  ×  10^14^ photons cm^−2^ per pulse, creating an initial carrier density of 7.5  ×  10^19^ carriers cm^−3^ in the hybrid sample.

## Conflict of Interest

The authors declare no conflict of interest.

## Author Contributions

S.P. and N.K. conceived and supervised the project. S.P., T.S., D.S., X.X., and P.A. performed ARPES measurements and analyzed the data, under supervision of N.K. and S.B. A.A., A.H., L.L., and V.T. prepared ML‐MoS_2_ samples. N.M., S.K., J.S., E.L.K., and S.B. performed TA and PL measurement and analyzed the data. S.P. and S.B. prepared the manuscript. All authors commented on the manuscript.

## Supporting information

Supporting Information

## Data Availability

The data that support the findings of this study are available from the corresponding author upon reasonable request.
