# Supplementary material for: Type‐I Energy Level Alignment at the PTCDA—Monolayer MoS2 Interface Promotes Resonance Energy Transfer and Luminescence Enhancement
Source: Adv Sci (Weinh). 2021 May 5;8(12):2100215. doi: 10.1002/advs.202100215 (PMC8224443; doi:10.1002/advs.202100215)
Supplement: Supplementary file 1 — Supporting Information [file ADVS-8-2100215-s001.pdf]

© 2021 Wiley-VCH GmbH

ADVANCED SCIENCE
Open Access

Supporting Information

for *Adv. Sci.,* DOI: 10.1002/advs.202100215

Type-I energy level alignment at the PTCDA - monolayer MoS_2 interface promotes resonance energy transfer and luminescence enhancement

Soohyung Park, Niklas Mutz, Sergey A. Kovalenko, Thorsten Schultz, Dongguen Shin, Areej Aljarb, Lain-Jong Li, Vincent Tung, Patrick Amsalem, Emil J. W. List-Kratochvil, Julia Stähler, Xiaomin Xu, Sylke Blumstengel, and Norbert Koch**

© Copyright 2020. WILEY-VCH GmbH.

Supporting Information

Type-I energy level alignment at the PTCDA - monolayer MoS_2 interface promotes resonance energy transfer and luminescence enhancement

Soohyung Park, Niklas Mutz, Sergey A. Kovalenko, Thorsten Schultz, Dongguen Shin, Areej Aljarb, Lain-Jong Li, Vincent Tung, Patrick Amsalem, Emil J. W. List-Kratochvil, Julia Stähler, Xiaomin Xu, Sylke Blumstengel, and Norbert Koch**

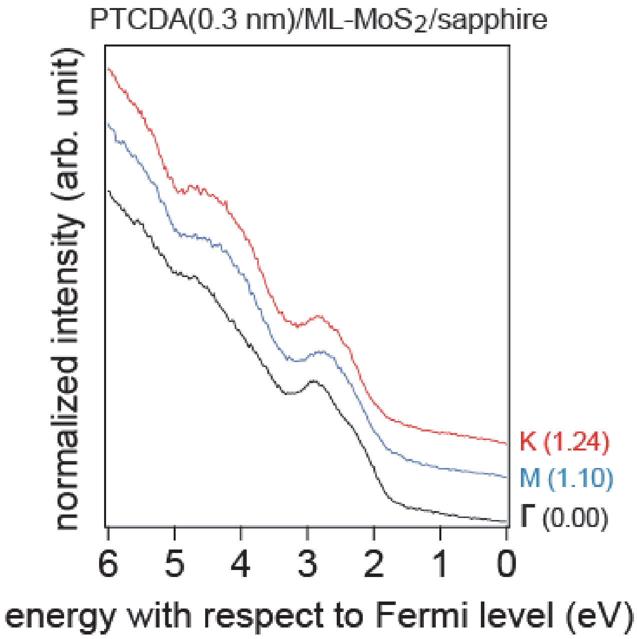

Figure S1. Angle-resolved photoemission spectroscopy (ARPES) of 0.3 nm PTCDA on monolayer MoS_2 measured at different angles, which shows no angular dependency.

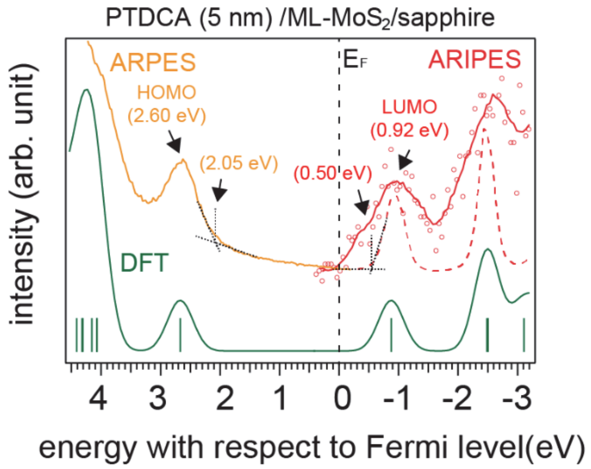

Figure S2. Angle-resolved photoemission spectroscopy (ARPES) and angle-resolved inverse photoemission spectroscopy (ARIPES) of PTCDA to determine the transport band gap (E_g) of PTCDA. The raw data of ARIPES and an average are plotted as red circles and solid line, respectively. A deconvoluted spectrum of ARIPES is plotted as dashed line to obtain the accurate onset value as detailed in previous work.[1] All spectra were recorded at the Γ point.

The combination of angle-resolved photoemission spectroscopy (ARPES) and angle-resolved inverse photoemission spectroscopy (ARIPES) were carried out to estimate the transport band gap (E_g) of PTCDA on monolayer (ML) MoS_2. The highest occupied molecular orbital (HOMO) and lowest unoccupied molecular orbital (LUMO) of PTCDA were estimated by onset of ARPES and ARIPES spectrum by 2.05 eV and 0.50 eV, respectively. The simple summation of HOMO and LUMO allow to determine the E_g of PTCDA by 2.55 eV, which is fair well agree with previous literatures.[2,3] Density function theory (DFT) calculation was performed using Gaussian software package.[4] Free standing model of PTCDA was used with hybrid functional of Lee-Yang-Parr (B3LYP) and 6-311 basis set.[5,6] To correct the under-estimated calculated E_g, the calculated spectra by DFT was rigidly shifted to match experimental spectra. In **Figure S1**, measured and calculated spectra were well matched, ensuring the reliability of determination of PTCDA E_g, particularly for ARIPES spectrum.

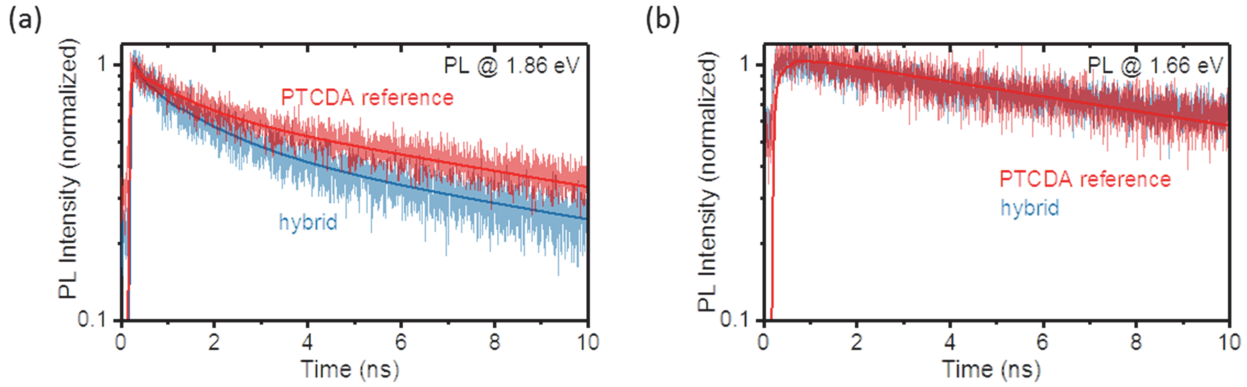

Figure S3. (a) and (b) PL transients of PTCDA in the PTCDA reference and hybrid sample recorded at 1.86 eV (a) and 1.66 eV (b). The fits were obtained by convoluting a triple-exponential decay law with the instrument response function. The PL in all experiments was excited at 2.21 eV. The temperature is 4.5 K.

Figure S3 shows photoluminescence (PL) transients of the hybrid and PTCDA reference samples recorded at 4.5 K at detection energies of 1.86 eV and 1.66 eV which correspond to the Y-band and E-band emission, respectively. The PL average lifetimes in the PTCDA reference samples are 6.4 ns (Y-band) and 22 ns (E-band). A shortening of the lifetime is only observed for the Y-band and an efficiency of RET of $\eta_{\mathrm{RET}} \approx 0.3$ is calculated following the procedure described in the main text.

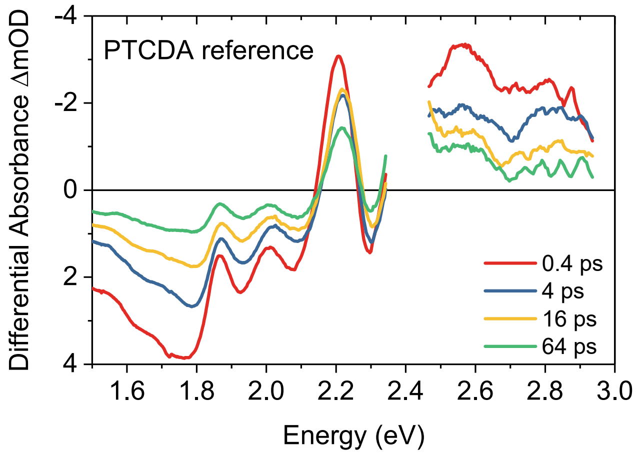

Figure S4. Transient absorption spectra of the PTCDA reference structure at different delay times. The data points in the spectral range of the pump pulse at 2.43 eV are removed.

Figure S4 shows transient absorption (TA) spectra of the PTCDA reference sample at different delay times. The TA spectra are superpositions of dispersive features due to the ML-MoS_2 excitonic A, B, and C resonances and of the photoinduced bleaching (PIB) and photoinduced absorption (PIA) of PTCDA. Compared with the TA spectra of the hybrid sample, the spectral weight is shifted towards the PTCDA features.

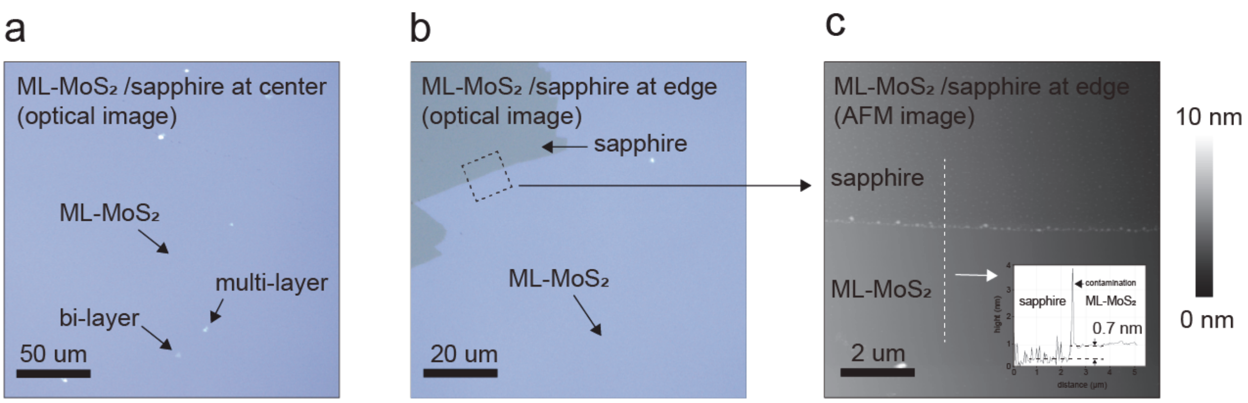

Figure S5. (a) Optical microscopy image with wide-scale at the center, (b) zoom-in image at the edge, and (c) AFM images at the edge of ML-MoS_2/sapphire. The line-cut shows the monolayer height (ca. 0.7 nm)

References

[1] S. Park, N. Mutz, T. Schultz, S. Blumstengel, A. Han, A. Aljarb, L.-J. Li, E. J. W. List-Kratochvil, P. Amsalem, N. Koch, *2D Mater.* **2018**, *5*, 025003.

[2] D. R. T. Zahn, G. N. Gavrila, M. Gorgoi, *Chem. Phys.* **2006**, *325*, 99.

[3] N. Sato, H. Yoshida, K. Tsutsumi, *J. Mater. Chem.* **1999**, *10*, 85.

[4] G. E. S. M. J. Frisch, G. W. Trucks, H. B. Schlegel, B. M. M. A. Robb, J. R. Cheeseman, G. Scalmani, V. Barone, H. P. H. G. A. Petersson, H. Nakatsuji, M.

Caricato, X. Li, M. H. A. F. Izmaylov, J. Bloino, G. Zheng, J. L. Sonnenberg, T. N. M. Ehara, K. Toyota, R. Fukuda, J. Hasegawa, M. Ishida, J. Y. Honda, O. Kitao, H. Nakai, T. Vreven, J. A. Montgomery, E. B. J. E. Peralta, F. Ogliaro, M. Bearpark, J. J. Heyd, J. N. K. N. Kudin, V. N. Staroverov, T. Keith, R. Kobayashi, J. T. K. Raghavachari, A. Rendell, J. C. Burant, S. S. Iyengar, J. B. C. M. Cossi, N. Rega, J. M. Millam, M. Klene, J. E. Knox, R. E. S. V. Bakken, C. Adamo, J. Jaramillo, R. Gomperts, J. W. O. O. Yazyev, A. J. Austin, R. Cammi, C. Pomelli, G. A. V. R. L. Martin, K. Morokuma, V. G. Zakrzewski, A. D. D. P. Salvador, J. J. Dannenberg, S. Dapprich, J. C. O. Farkas, J. B. Foresman, J. V. Ortiz, and D. J. Fox, *Gaussian 09, Revis. B.01, Gaussian Inc., Wallingford, CT* **2009**.

[5] A. D. Becke, *J. Chem. Phys.* **1993**, *98*, 5648.

[6] C. Lee, W. Yang, R. G. Parr, *Phys. Rev. B* **1988**, *37*, 785.
